# Supplementary material for: The variable selection of two-part regression model for semicontinuous data
Source: PLoS One. 2025 Jun 3;20(6):e0322937. doi: 10.1371/journal.pone.0322937 (PMC12132987; doi:10.1371/journal.pone.0322937)
Supplement: S1 Appendix — (DOCX) [file pone.0322937.s001.docx]

**Appendix for “The Variable Selection of Two-part Regression Model for Semicontinuous Data” by Yahui Lu^1^, Aiyi Liu^2^, Tao Jiang^3^***

^1^School of Economics and Management, Zhejiang University of Science and Technology, Hangzhou 310023, China

^2^Biostatistics and Bioinformatics Branch, Eunice Kennedy Shriver National Institute of Child Health and Human Development, Bethesda, MD 20817, USA

^3^School of Statistics and Mathematics, Zhejiang Gongshang University, Hangzhou 310018, China

^*^Correspondence: [jtao@263.net](mailto:jtao@263.net)

**S1 Varying Zero Proportions including low, moderate, and high zero proportions.**

In the simulation, covariables are generated from the multivariate normal distribution$N_{q}(0,\Sigma)$, where covariance matrix is Σ whose elements are $\rho^{|i-j|}(i,j=1,\ldots,q)$. In the section, we set $\rho=0.6$, $q=15$, and the sample size is $n=300$. According to the Bernoulli-Normal regression model, the real model is assumed as follows , where $\text{logit}(\pi_{i})=\mathbf{z}_{1}^{T}\mathbf{\beta}_{1}$, $\mu_{i}=\mathbf{z}_{2}^{T}\mathbf{\beta}_{2}$. We set $\mathbf{z}_{1}=\mathbf{z}_{2}$ in the following simulation scenarios, and each scenario is repeated 500 times. In this section, the model coefficient settings include different zero proportions, including low, moderate, and high zero proportions.

1. When $\Pr(\beta=0)=0.3$, the regression coefficients in model are set as

$$\mathbf{\beta}_{1}=(0.2,0.30,1,0.8,0.4,-0.2,0,0,0.6,0,0,0,0.8,0.7,1.2,-1)^{T}\text{,}$$

$$\mathbf{\beta}_{2}=(0.1,1.10,1,-0.36,0.6,0,0,0,0.8,0,0,0.8,0.7,1.2,-1,0.8)^{T}\text{;}$$

1. When $\Pr(\beta=0)=0.6$, the regression coefficients in model are set as

$$\mathbf{\beta}_{1}=(0.2,0.30,1,0.8,0.4,-0.2,0,0,0.6,0,0,0,0,0,0,0)^{T}\text{,}$$

$$\mathbf{\beta}_{2}=(0.1,1.10,1,-0.36,0.6,0,0,0,0.8,0,0,0,0,0,0,0)^{T}\text{;}$$

1. When $\Pr(\beta=0)=0.8$, the regression coefficients in model are set as

$\mathbf{\beta}_{1}=(0.2,0.30,1,0,0.4,0,0,0,0,0,0,0,0,0,0,0)^{T}$,

$$\mathbf{\beta}_{2}=(0.1,1.10,1,-0.36,0,0,0,0,0,0,0,0,0,0,0,0)^{T}.$$

Table S1-S3 below present the simulation results. From the tables, the two proposed methods have a good variable selection effect for different zero proportions, and the adaptive Lasso method is superior to Lasso method.

**Table S1.** Simulation results of the setting $\Pr(\beta=0)=0.3$

| **Method** | **MPSE** | **MSE** | **Sensitivity** | **Specificity** | **Accuracy** |
| --- | --- | --- | --- | --- | --- |
| Binomial Part |  |  |  |  |  |
| LASSO | 0.7982 | 0.0552 | 0.9332 | 0.4636 | 0.7767 |
| ALASSO | 0.7799 | 0.0605 | 0.8450 | 0.7448 | 0.8116 |
| Normal Part |  |  |  |  |  |
| LASSO | 1.1203 | 0.0149 | 0.9970 | 0.2960 | 0.7633 |
| ALSSO | 1.0888 | 0.0134 | 0.9902 | 0.7244 | 0.9016 |
| Two Parts |  |  |  |  |  |
| LASSO | 1.9184 | 0.0350 | 0.9651 | 0.3798 | 0.7700 |
| ALSSO | 1.8686 | 0.0370 | 0.9176 | 0.7346 | 0.8566 |

**Table S2.** Simulation results of the setting $\Pr(\beta=0)=0.6$

| **Method** | **MPSE** | **MSE** | **Sensitivity** | **Specificity** | **Accuracy** |
| --- | --- | --- | --- | --- | --- |
| Binomial Part |  |  |  |  |  |
| LASSO | 1.0906 | 0.0123 | 0.9644 | 0.5122 | 0.6629 |
| ALASSO | 1.0382 | 0.0088 | 0.9784 | 0.7754 | 0.8431 |
| Normal Part |  |  |  |  |  |
| LASSO | 0.9359 | 0.0265 | 0.8433 | 0.6469 | 0.7255 |
| ALSSO | 0.9235 | 0.0337 | 0.7517 | 0.7842 | 0.7712 |
| Two Parts |  |  |  |  |  |
| LASSO | 2.0265 | 0.0194 | 0.8984 | 0.5760 | 0.6942 |
| ALSSO | 1.9617 | 0.0213 | 0.8547 | 0.7796 | 0.8071 |

**Table S3.** Simulation results of the setting $\Pr(\beta=0)=0.8$

| **Method** | **MPSE** | **MSE** | **Sensitivity** | **Specificity** | **Accuracy** |
| --- | --- | --- | --- | --- | --- |
| Binomial Part |  |  |  |  |  |
| LASSO | 1.1083 | 0.0129 | 0.9393 | 0.7367 | 0.7772 |
| ALASSO | 1.0895 | 0.0183 | 0.8160 | 0.8135 | 0.8140 |
| Normal Part |  |  |  |  |  |
| LASSO | 1.0628 | 0.0080 | 0.9707 | 0.6285 | 0.6969 |
| ALSSO | 1.0240 | 0.0064 | 0.9740 | 0.8098 | 0.8427 |
| Two Parts |  |  |  |  |  |
| LASSO | 2.1711 | 0.0104 | 0.9550 | 0.6826 | 0.7371 |
| ALSSO | 2.1135 | 0.0124 | 0.8950 | 0.8117 | 0.8283 |

**S2 Different Variable-Effect Strengths including strong and weak signal between predictors and outcome in one or both parts of the model.**

In linear regression, it is necessary to check the effect strength of different variables (independent variables) on dependent variables, and the regression coefficient can directly reflect the influence degree of independent variables on dependent variables. If the regression coefficient is positive, it means that the dependent variable increases with the increase of the independent variable. If the regression coefficient is negative, it means that the dependent variable decreases as the independent variable increases. The greater the absolute value of the regression coefficient, the more significant the influence of the independent variable on the dependent variable‌.

To observe different Variable-Effect strengths including strong and weak signal between predictors and outcome, we conduct simulation studies by setting different regression coefficient values, so as to further verify the variable selection effect of our proposed methods in the Bernoulli-Normal model. In the study, covariables are generated from the multivariate normal distribution $N_{q}(0,\Sigma)$, where covariance matrix is Σ whose elements are $\rho^{|i-j|}(i,j=1,\ldots,q)$. In the settings above, the sample size is $n=300$, $\rho=0,0.6$, $q=15$. we set $\mathbf{z}_{1}=\mathbf{z}_{2}$ in the following simulation scenarios.

1. When there is a weak Variable-Effect strength between the predictor and the outcome, the regression coefficients are set to

$$\mathbf{\beta}_{1}=(0.2,0.2,0.2,0.2,0.2,-0.2,0,0,0.2,0,0,0,0,0,0,0)^{T}\text{，}$$

$$\mathbf{\beta}_{2}=(0.1,0.1,0.1,-0.1,0.1,0,0,0,0.1,0,0,0,0,0,0,0)^{T}\text{；}$$

1. When there is a strong Variable-Effect strength between the predictor and the outcome, the regression coefficients are set to

$$\mathbf{\beta}_{1}=(1.2,1.2,1.2,1.2,1.2,-1.2,0,0,1.2,0,0,0,0,0,0,0)^{T}\text{,}$$

$$\mathbf{\beta}_{2}=(1,1,1,-1,1,0,0,0,1,0,0,0,0,0,0,0)^{T}.$$

Table S4 - S7 below present the simulation results. From the Table S4 and Table S5, the proposed methods have high Specificity value and low Sensitivity value. This is because the strength of the weak variable effect between the predictor and the outcome results in more variable coefficients being estimated at zero. On the contrary, from the Table S6 and Table S7, the proposed methods have high Sensitivity value and low Specificity value, with the Sensitivity value mostly approaching 1. This is because the strength of the strong variable effect between the predictor and the outcome results in more variable coefficients being estimated at nonzero, thus classifying more variables as important. However, all the results show that the proposed adaptive Lasso method has higher Sensitivity value, Specificity value and Accuracy value than the Lasso method. Therefore, the adaptive lasso method has a better variable selection effect for the Bernoulli-Normal model.

**Table S4.** Simulation results of the weak Variable-Effect ($\rho=0$)

| **Method** | **MPSE** | **MSE** | **Sensitivity** | **Specificity** | **Accuracy** |
| --- | --- | --- | --- | --- | --- |
| Binomial Part |  |  |  |  |  |
| LASSO | 1.3638 | 0.0105 | 0.6113 | 0.7227 | 0.6781 |
| ALASSO | 1.3501 | 0.0111 | 0.6537 | 0.7622 | 0.7188 |
| Normal Part |  |  |  |  |  |
| LASSO | 1.0295 | 0.0034 | 0.3512 | 0.8128 | 0.6589 |
| ALSSO | 1.0201 | 0.0043 | 0.4256 | 0.7902 | 0.6687 |
| Two Parts |  |  |  |  |  |
| LASSO | 2.3923 | 0.0070 | 0.4931 | 0.7701 | 0.6685 |
| ALSSO | 2.3703 | 0.0077 | 0.5500 | 0.7769 | 0.6937 |

**Table S5.** Simulation results of the weak Variable-Effect ($\rho=0.6$)

| **Method** | **MPSE** | **MSE** | **Sensitivity** | **Specificity** | **Accuracy** |
| --- | --- | --- | --- | --- | --- |
| Binomial Part |  |  |  |  |  |
| LASSO | 1.3281 | 0.0125 | 0.5080 | 0.7808 | 0.6717 |
| ALASSO | 1.3186 | 0.0178 | 0.6130 | 0.7604 | 0.7015 |
| Normal Part |  |  |  |  |  |
| LASSO | 1.0353 | 0.0037 | 0.3528 | 0.8064 | 0.6552 |
| ALSSO | 1.0138 | 0.0052 | 0.3072 | 0.8374 | 0.6607 |
| Two Parts |  |  |  |  |  |
| LASSO | 2.3634 | 0.0081 | 0.4375 | 0.7943 | 0.6635 |
| ALSSO | 2.3325 | 0.0115 | 0.4740 | 0.8009 | 0.6811 |

**Table S6.** Simulation results of the strong Variable-Effect ($\rho=0$)

| **Method** | **MPSE** | **MSE** | **Sensitivity** | **Specificity** | **Accuracy** |
| --- | --- | --- | --- | --- | --- |
| Binomial Part |  |  |  |  |  |
| LASSO | 1.7752 | 0.0386 | 1.0000 | 0.3867 | 0.6321 |
| ALASSO | 1.7580 | 0.0257 | 1.0000 | 0.8264 | 0.8959 |
| Normal Part |  |  |  |  |  |
| LASSO | 1.0935 | 0.0064 | 1.0000 | 0.4880 | 0.6587 |
| ALSSO | 1.0454 | 0.0043 | 1.0000 | 0.8392 | 0.8928 |
| Two Parts |  |  |  |  |  |
| LASSO | 1.8686 | 0.0225 | 1.0000 | 0.4401 | 0.6454 |
| ALSSO | 1.8034 | 0.0150 | 1.0000 | 0.8332 | 0.8943 |

**Table S7.** Simulation results of the strong Variable-Effect ($\rho=0.6$)

| **Method** | **MPSE** | **MSE** | **Sensitivity** | **Specificity** | **Accuracy** |
| --- | --- | --- | --- | --- | --- |
| Binomial Part |  |  |  |  |  |
| LASSO | 0.6729 | 0.0621 | 1.0000 | 0.4816 | 0.6889 |
| ALASSO | 0.6632 | 0.0527 | 0.9993 | 0.7796 | 0.8675 |
| Normal Part |  |  |  |  |  |
| LASSO | 1.0809 | 0.0113 | 1.0000 | 0.4836 | 0.6557 |
| ALSSO | 1.0404 | 0.0077 | 1.0000 | 0.8240 | 0.8827 |
| Two Parts |  |  |  |  |  |
| LASSO | 1.7538 | 0.0367 | 1.0000 | 0.4826 | 0.6723 |
| ALSSO | 1.7037 | 0.0302 | 0.9996 | 0.8029 | 0.8751 |

**S3 Different Correlations Between Parts. The current simulation assumes independent parts. However, it will be useful to test the robustness of the model under the violation of the independence assumption.**

In this paper, we seek a feasible way of conducting variable selection for Bernoulli-Normal model. Based on the basic idea of constructing a two-part model, we divide the model into two parts. For the first part, whether *X* is zero can be treated as being from a Bernoulli distribution, that is, it is assumed that *Y* follows a Bernoulli distribution; For the second part, the nonzero part is assumed to follow a normal distribution. The model assumes that the two parts are independent. But when the two parts do not satisfy the independence, then the model becomes a random effects Bernoulli-Normal model, that is

$f(x_{i})=(1-\pi_{i})^{1-y_{i}}[\pi_{i}N(x_{i};\mu_{i},\sigma^{2})]^{y_{i}}$,

$\text{logit}(\pi_{i})=\text{logit}[\text{Pr}(X_{i}>0)]=\mathbf{z}_{1i}^{T}\mathbf{\beta}_{1}+u_{1i}$,

$\mu_{i}=\text{E}[X_{i}|X_{i}>0]=\mathbf{z}_{2i}^{T}\mathbf{\beta}_{2}+u_{2i}$.

The random effects $u_{1i}$ and $u_{2i}$ from the two parts are assumed to be jointly normally distributed and possibly correlated

$\left(\begin{matrix} u_{1i} \\ u_{2i} \end{matrix}\right)\sim N\left(\left[\begin{matrix} 0 \\ 0 \end{matrix}\right],\left[\begin{matrix} \sigma_{1} & \sigma_{12} \\ \sigma_{12} & \sigma_{2} \end{matrix}\right]\right)$.

In this section, in order to verify the variable selection effect of our proposed methods in the random effects Bernoulli-Normal model, we conduct simulation studies to examine the finite sample performance of the model. In the study, covariables are generated from the multivariate normal distribution $N_{q}(0,\Sigma)$, where covariance matrix is Σ whose elements are $\rho^{|i-j|}(i,j=1,\ldots,q)$. In the settings above, the sample size is $n=300$, $\rho=0,0.6$, $q=15$, $\left(\sigma_{1},\sigma_{2},\sigma_{12}\right)=\left(1,1.44,0.6\right)$. In the following simulation scenarios, we set $\mathbf{z}_{1}=\mathbf{z}_{2}$ and the regression coefficients in model are set as

$$\mathbf{\beta}_{1}=(0.2,0.30,1,0.8,0.4,-0.2,0,0,0.6,0,0,0,0,0,0,0)^{T}\text{,}$$

$$\mathbf{\beta}_{2}=(0.1,1.10,1,-0.36,0.6,0,0,0,0.8,0,0,0,0,0,0,0)^{T}.$$

Table S8 and Table S9 below present the simulation results. From the tables, under the violation of the independence assumption between parts, the two proposed methods also have good effect for the variable selection of the random effects Bernoulli-Normal model, and the adaptive Lasso method is superior to Lasso method. However, compared with the Bernoulli-Normal model, the proposed methods have higher prediction error in the random effects Bernoulli-Normal model. Because fitting a random effects two-part model itself faces substantial computational challenges, which mostly arise from numerical integration with respect to correlated random effects.

**Table S8.** Simulation results of the random effects Bernoulli-Normal model

($\rho=0$)

| **Method** | **MPSE** | **MSE** | **Sensitivity** | **Specificity** | **Accuracy** |
| --- | --- | --- | --- | --- | --- |
| Binomial Part |  |  |  |  |  |
| LASSO | 1.1802 | 0.0313 | 0.9067 | 0.5736 | 0.7068 |
| ALASSO | 1.1649 | 0.0253 | 0.8390 | 0.7902 | 0.8097 |
| Normal Part |  |  |  |  |  |
| LASSO | 1.1395 | 0.0136 | 0.9992 | 0.4868 | 0.6576 |
| ALSSO | 2.0469 | 0.0106 | 0.9944 | 0.7840 | 0.8541 |
| Two Parts |  |  |  |  |  |
| LASSO | 3.3197 | 0.0225 | 0.9487 | 0.5279 | 0.6822 |
| ALSSO | 3.2118 | 0.0180 | 0.9096 | 0.7869 | 0.8319 |

**Table S9.** Simulation results of the random effects Bernoulli-Normal model ($\rho=0.6$)

| **Method** | **MPSE** | **MSE** | **Sensitivity** | **Specificity** | **Accuracy** |
| --- | --- | --- | --- | --- | --- |
| Binomial Part |  |  |  |  |  |
| LASSO | 1.0367 | 0.0367 | 0.8140 | 0.6573 | 0.7200 |
| ALASSO | 1.0200 | 0.0371 | 0.7120 | 0.7960 | 0.7624 |
| Normal Part |  |  |  |  |  |
| LASSO | 2.1315 | 0.0239 | 0.9364 | 0.5440 | 0.6748 |
| ALSSO | 2.0363 | 0.0201 | 0.9456 | 0.7590 | 0.8212 |
| Two Parts |  |  |  |  |  |
| LASSO | 3.1682 | 0.0303 | 0.8696 | 0.5977 | 0.6974 |
| ALSSO | 3.0563 | 0.0286 | 0.8182 | 0.7765 | 0.7918 |

**S4 The model performance with high-dimensional data**

We conduct more simulations by generated covariables from the multivariate normal distribution $N_{q}(0,\Sigma)$, where covariance matrix is $\Sigma$ whose elements are$\rho^{|i-j|}(i,j=1,\ldots,q)$ with $q=100, \rho=0.6$, and total sample sizes is $n=300$. In the following simulation scenario, we set $\mathbf{z}_{1}=\mathbf{z}_{2}$ and the regression coefficients are set as

$$\begin{matrix} \mathbf{\beta}_{1}=(0.2,0.30,1,0.8,0.4,-0.2,0,0,0.6,0,0,0,0,0,0,0,1.1,-0.2,0,0,0,0,0,0.6,0,0\text{，} \\ \text{ }0.30,1,0.8,0.4,-0.2,0,0,0.6,0,0,0,0,0,0,0,1.1,-0.2,0,0,0,0,0,0.6,0,0\text{，} \\ \text{ }0.30,1,0.8,0.4,-0.2,0,0,0.6,0,0,0,0,0,0,0,1.1,-0.2,0,0,0,0,0,0.6,0,0\text{，} \\ \text{ }0.30,1,0.8,0.4,-0.2,0,0,0.6,0,0,0,0,0,0,0,1.1,-0.2,0,0,0,0,0,0.6,0,0)^{T}\text{,} \end{matrix}$$

$$\begin{matrix} \mathbf{\beta}_{2}=(0.1,1.10,1,-0.36,0.6,0,0,0,0.8,0,0,0,0,0,0,0,0.9,0.7,0,0,0,0,0,1.2,0,0\text{，} \\ \text{ }1.10,1,-0.36,0.6,0,0,0,0.8,0,0,0,0,0,0,0,0.9,0.7,0,0,0,0,0,1.2,0,0\text{，} \\ \text{ }1.10,1,-0.36,0.6,0,0,0,0.8,0,0,0,0,0,0,0,0.9,0.7,0,0,0,0,0,1.2,0,0\text{，} \\ \text{ }1.10,1,-0.36,0.6,0,0,0,0.8,0,0,0,0,0,0,0,0.9,0.7,0,0,0,0,0,1.2,0,0)^{T} \end{matrix}$$

**Table S10.** Simulation results of the high-dimensional setting

| **Method** | **MPSE** | **MSE** | **Sensitivity** | **Specificity** | **Accuracy** |
| --- | --- | --- | --- | --- | --- |
| Binomial Part |  |  |  |  |  |
| LASSO | 0.6987 | 0.0583 | 0.7361 | 0.6773 | 0.6985 |
| ALASSO | 0.6187 | 0.0833 | 0.7354 | 0.7026 | 0.7174 |
| Normal Part |  |  |  |  |  |
| LASSO | 2.0063 | 0.0165 | 0.9800 | 0.4847 | 0.6432 |
| ALSSO | 1.3562 | 0.0151 | 0.9783 | 0.6842 | 0.7769 |
| Two Parts |  |  |  |  |  |
| LASSO | 2.7050 | 0.0374 | 0.8509 | 0.5781 | 0.6708 |
| ALSSO | 1.9750 | 0.0492 | 0.8346 | 0.6931 | 0.7276 |

The results are repeated 500 times and presented in the Table S10 above. Results from table demonstrate that the adaptive Lasso method performs better than Lasso method for the Bernoulli-Normal model. In addition, our research is mainly for the case of $p\ll n$, when $p\gg n$, the coordinate descent method may take more time to estimate parameters, which is also one of the contents we are currently studying.

**S5 Simulate scenarios where** **some predictors only influence the zero part, while others only influence the non-zero part. This will test the method's capacity to select variables that are important for only one part of the model.**

The current simulation assumes the same covariates for both parts of the model. But some predictors only influence the zero part, and others only influence the non-zero part. In order to simulate the scenarios, we generate data with different covariables of two parts. The covariables of the zero part ($\mathbf{z}_{1}$) are generated from the multivariate normal distribution $N_{15}(0,\Sigma)$, and the covariables of the non-zero part ($\mathbf{z}_{2}$) are generated from the multivariate normal distribution $N_{10}(0,\Sigma)$, where covariance matrix is $\Sigma$ whose elements are$\rho^{|i-j|}(i,j=1,\ldots,q)$. In the settings above, the sample size is $n=300$, $\rho=0,0.6$, and the regression coefficients in model are set as

$\mathbf{\beta}_{1}=(0.2,0.30,1,0.8,0.4,-0.2,0,0,0.6,0,0,0,0,0,0,0)^{T}$;

$\mathbf{\beta}_{2}=(0.1,1.10,1,-0.36,0.6,0,0,0,0,0,0)^{T}$.

The results are repeated 500 times and presented in the Table S11-S12 below. The results show that when the covariables of the two parts are different, the proposed methods still have good variable selection effect in the Bernoulli-Normal model. And the adaptive Lasso method performs better than the Lasso method.

**Table S11.** Simulation results of the different covariables setting ($\rho=0$)

| **Method** | **MPSE** | **MSE** | **Sensitivity** | **Specificity** | **Accuracy** |
| --- | --- | --- | --- | --- | --- |
| Binomial Part |  |  |  |  |  |
| LASSO | 1.0904 | 0.0174 | 0.9400 | 0.5227 | 0.6896 |
| ALASSO | 1.0724 | 0.0160 | 0.8760 | 0.7687 | 0.8116 |
| Normal Part |  |  |  |  |  |
| LASSO | 1.0715 | 0.0058 | 0.1000 | 0.4990 | 0.6994 |
| ALSSO | 1.0353 | 0.0047 | 0.9990 | 0.8143 | 0.8882 |
| Two Parts |  |  |  |  |  |
| LASSO | 2.1619 | 0.0127 | 0.9640 | 0.5132 | 0.6935 |
| ALSSO | 2.1076 | 0.0115 | 0.9252 | 0.7869 | 0.8422 |

**Table S12.** Simulation results of the different covariables setting ($\rho=0.6$)

| **Method** | **MPSE** | **MSE** | **Sensitivity** | **Specificity** | **Accuracy** |
| --- | --- | --- | --- | --- | --- |
| Binomial Part |  |  |  |  |  |
| LASSO | 0.9378 | 0.0255 | 0.8430 | 0.6698 | 0.7391 |
| ALASSO | 0.9238 | 0.0345 | 0.7510 | 0.7851 | 0.7715 |
| Normal Part |  |  |  |  |  |
| LASSO | 1.0667 | 0.0135 | 0.9555 | 0.4727 | 0.6658 |
| ALSSO | 1.0315 | 0.0109 | 0.9680 | 0.7540 | 0.8396 |
| Two Parts |  |  |  |  |  |
| LASSO | 2.0045 | 0.0207 | 0.8880 | 0.5909 | 0.7098 |
| ALSSO | 1.9552 | 0.0250 | 0.8378 | 0.7727 | 0.7987 |

**S6 Simulate cases where the model is mis-specified for one or both parts, to test how robust the variable selection method is to model misspecification.**

The paper mainly discusses the variable selection problem of semicontinuous data, characterized by a sizable number of zeros and observations from a continuous distribution. We propose the Lasso and the adaptive Lasso methods for the variable selection of Bernoulli-Normal regression model. The presence/absence of zeros is assumed to follow a Bernoulli distribution. The nonzero values are assumed to follow a normal distribution. When the continuous part of the distribution happens to be non-normal, the variable selection performance of the Lasso and the adaptive Lasso methods may be affected to some extent. At this time, the proposed methods can be extended or improved by making reasonable assumptions about the distribution of continuous data. This is also one of our future research directions.

When the continuous part of the distribution happens to be non-normal, we further consider the continuous part to follow gamma distributions. At this point, we still use the Bernoulli-Normal model to fit data. To evaluate the performance of the relative performance of the Lasso and the adaptive Lasso methods, the gamma distribution we used is induced by G$(\alpha, \beta)$. In the simulation, we only generate the gamma distributions with $\beta=1$, and total sample sizes is $n=300$. Covariables are generated from the multivariate normal distribution$N_{q}(0,\Sigma)$, where covariance matrix is Σ whose elements are $\rho^{|i-j|}(i,j=1,\ldots,q)$ with $\rho=0,0.6$, $q=15$. Then the real data is generated by , where $\text{logit}(\pi_{i})=\mathbf{z}_{1}^{T}\mathbf{\beta}_{1}$, $\log\alpha_{i}=\mathbf{z}_{2}^{T}\mathbf{\beta}_{2}$. We set covariables $\mathbf{z}_{1}=\mathbf{z}_{2}$, and the regression coefficients in model are set as

$\mathbf{\beta}_{1}=(0.2,0.30,1,0.8,0.4,-0.2,0,0,0.6,0,0,0,0,0,0,0)^{T}$;

$\mathbf{\beta}_{2}=(0.1,1.10,1,-0.36,0.6,0,0,0,0.8,0,0,0,0,0,0,0)^{T}$.

**Table S13.** Simulation results of the continuous part follow gamma distributions ($\rho=0$)

| **Method** | **MPSE** | **MSE** | **Sensitivity** | **Specificity** | **Accuracy** |
| --- | --- | --- | --- | --- | --- |
| Binomial Part |  |  |  |  |  |
| LASSO | 1.0910 | 0.0168 | 0.9437 | 0.5224 | 0.6909 |
| ALASSO | 1.0767 | 0.0163 | 0.8790 | 0.7791 | 0.8191 |
| Normal Part |  |  |  |  |  |
| LASSO | 106.1605 | 0.6396 | 0.9500 | 0.5224 | 0.6649 |
| ALSSO | 107.1314 | 0.9241 | 0.9200 | 0.7730 | 0.8220 |
| Two Parts |  |  |  |  |  |
| LASSO | 107.2515 | 0.3282 | 0.9465 | 0.5224 | 0.6779 |
| ALSSO | 108.2081 | 0.4702 | 0.8976 | 0.7759 | 0.8205 |

**Table S14.** Simulation results of the continuous part follow gamma distributions ($\rho=0.6$)

| **Method** | **MPSE** | **MSE** | **Sensitivity** | **Specificity** | **Accuracy** |
| --- | --- | --- | --- | --- | --- |
| Binomial Part |  |  |  |  |  |
| LASSO | 0.9383 | 0.0266 | 0.8510 | 0.6536 | 0.7325 |
| ALASSO | 0.9190 | 0.0334 | 0.7547 | 0.7816 | 0.7708 |
| Normal Part |  |  |  |  |  |
| LASSO | 64.0235 | 0.2807 | 0.8772 | 0.5738 | 0.6749 |
| ALSSO | 58.4421 | 0.4029 | 0.8652 | 0.7702 | 0.8019 |
| Two Parts |  |  |  |  |  |
| LASSO | 64.9618 | 0.1536 | 0.8629 | 0.6116 | 0.7037 |
| ALSSO | 59.3611 | 0.2182 | 0.8049 | 0.7756 | 0.7863 |

When the continuous part of the actual data follows gamma distributions, but we still use the mis-specified Bernoulli-Normal model to fit. Table S13-S14 present the results of 500 repetitions. The results show that the proposed methods still have good variable selection effect, and the adaptive Lasso method is superior to the Lasso method. But all the proposed methods have high prediction error. This is not surprising since the non-zero part is not given a suitable model to fit.

**S7 R program code**

**1. Simulation section (Lasso variable selection)**

library(MASS)

library(Matrix)

library(foreach)

library("glmnet")

n# The sample size is set to 100,300

sd # The Variance is set to 1

p # The variable dimensions are set to 10,15,25

Rho # The correlation coefficient is set to 0,0.6

Beta #$\mathbf{\beta}$

Gamma #$\mathbf{\gamma}$

Sigma=matrix(rep(0,p*p),p,p)

nBeta<-which(Beta!=0)

nGamma<-which(Gamma!=0)

zBeta<-which(Beta==0)

zGamma<-which(Gamma==0)

for(i in 1:p)

{for(j in 1:p)

{Sigma[i,j]=Rho^(abs(i-j))}

}

TSPE1=0

TSE1=0

TSen1=0

TSpe1=0

TAcc1=0

TSPE2=0

TSE2=0

TSen2=0

TSpe2=0

TAcc2=0

TSPE=0

TSE=0

TSen=0

TSpe=0

TAcc=0

for(se in 1:500)

{x1=mvrnorm(n, rep(0,p), Sigma)

#x1=scale(x1,center=T,scale=T)

mu = x1%*%t(t(Beta))

#mu=exp(mu)

y1=rnorm(n,mu,sd)

linbin=x1%*%t(t(Gamma))

pro = exp(linbin)/(1+exp(linbin))

y2=rbinom(n,1,pro)

y=y1*y2

xn=x1[which(y!=0),]

yn=y[which(y!=0)]

fit1 = glmnet(xn, yn,family='gaussian')

#print(fit1)

#plot(fit1)

cv.fit1=cv.glmnet(xn,yn,family='gaussian',type.measure="mse")

#plot(cv.fit1)

cv.fit1$lambda.min # Optimal lambda value

SPE1=min(cv.fit1$cvm) # Minimum prediction mean square error

TSPE1=SPE1+TSPE1

#cv.fit1$lambda.1se#The lambda value of the simplest model within one standard deviation of lambda.min.

coefficients1<-coef(fit1,s=cv.fit1$lambda.min)

Active.Index1<-which(coefficients1!=0) # Feature index whose coefficient is not 0

nActive1=Active.Index1-1

Active.coefficients1<-coefficients1[Active.Index1] # Eigencoefficient values whose coefficients are not zero

SEn1=sum((Beta[nActive1[-1]]-Active.coefficients1[-1])^2)

zActive.Index1<-which(coefficients1==0) # Feature index whose coefficient is not 0

zActive1<-zActive.Index1-1

SEz1=sum((Beta[zActive1])^2)

SE1=(SEn1+SEz1)/p

TSE1=TSE1+SE1

ln1=intersect(nBeta, nActive1)

lz1=intersect(zBeta, zActive1)

Sen1=length(ln1)/length(nBeta)

Spe1=length(lz1)/length(zBeta)

TSen1=Sen1+TSen1

TSpe1=Spe1+TSpe1

Acc1=(length(ln1)+length(lz1))/p

TAcc1=Acc1+TAcc1

fit2 = glmnet(x1, y2,family='binomial')

#print(fit2)

#plot(fit2)

cv.fit2=cv.glmnet(x1,y2,family='binomial',type.measure="deviance")

#plot(cv.fit2)

cv.fit2$lambda.min # Optimal lambda value

SPE2=min(cv.fit2$cvm)# Minimum prediction mean square error

TSPE2=SPE2+TSPE2

#cv.fit1$lambda.1se# The lambda value of the simplest model within one standard deviation of lambda.min.

coefficients2<-coef(fit2,s=cv.fit2$lambda.min)

Active.Index2<-which(coefficients2!=0) # Feature index whose coefficient is not 0

nActive2=Active.Index2-1

Active.coefficients2<-coefficients2[Active.Index2] # Eigencoefficient values whose coefficients are not zero

SEn2=sum((Gamma[nActive2[-1]]-Active.coefficients2[-1])^2)

zActive.Index2<-which(coefficients2==0) # Feature index with coefficient 0

zActive2=zActive.Index2-1

SEz2=sum((Gamma[zActive2])^2)

SE2=(SEn2+SEz2)/p

TSE2=SE2+TSE2

ln2=intersect(nGamma, nActive2)

lz2=intersect(zGamma, zActive2)

Sen2=length(ln2)/length(nGamma)

Spe2=length(lz2)/length(zGamma)

TSen2=Sen2+TSen2

TSpe2=Spe2+TSpe2

Acc2=(length(ln2)+length(lz2))/p

TAcc2=Acc2+TAcc2

SPE=SPE1+SPE2

TSPE=SPE+TSPE

SE=(SE1+SE2)/2

TSE=SE+TSE

Sen=(length(ln1)+length(ln2))/(length(nBeta)+length(nGamma))

TSen=Sen+TSen

Spe=(length(lz1)+length(lz2))/(length(zBeta)+length(zGamma))

TSpe=Spe+TSpe

Acc=(length(ln1)+length(ln2)+length(lz1)+length(lz2))/(p+p)

TAcc=Acc+TAcc

}

TSPE1/500 # The prediction mean square error of Continuous part

TSE1/500 # Mean square error of continuously distributed parameters

TSen1/500 # Sensitivity of continuous distribution

TSpe1/500# Specificity of continuous distribution

TAcc1/500# Accuracy of continuous parts

TSPE2/500# Prediction mean square error of binomial part

TSE2/500# Mean square error of continuously distributed parameters

TSen2/500# Sensitivity of continuous distribution

TSpe2/500# Specificity of continuous distribution

TAcc2/500# Accuracy of continuous parts

TSPE/500# the prediction mean square error of the two parts

TSE/500#Mean square error of two parts

TSen/500# sensitivity of two parts

TSpe/500# specificity of two parts

TAcc/500# accuracy of two parts

**2. Simulation part (ALasso variable selection)**

library(MASS)

library(Matrix)

library(foreach)

library("glmnet")

n# 100，300

sd # 1

p # 10，15，25

Rho # 0，0.6

Beta #$\mathbf{\beta}$

Gamma #$\mathbf{\gamma}$

Sigma=matrix(rep(0,p*p),p,p)

nBeta<-which(Beta!=0)

nGamma<-which(Gamma!=0)

zBeta<-which(Beta==0)

zGamma<-which(Gamma==0)

for(i in 1:p)

{for(j in 1:p)

{Sigma[i,j]=Rho^(abs(i-j))}

}

TSPE1=0

TSE1=0

TSen1=0

TSpe1=0

TAcc1=0

TSPE2=0

TSE2=0

TSen2=0

TSpe2=0

TAcc2=0

TSPE=0

TSE=0

TSen=0

TSpe=0

TAcc=0

for(se in 1:500)

{x1=mvrnorm(n, rep(0,p), Sigma)

#x1=scale(x1,center=T,scale=T)

mu = x1%*%t(t(Beta))

#mu=exp(mu)

y1=rnorm(n,mu,sd)

linbin=x1%*%t(t(Gamma))

pro = exp(linbin)/(1+exp(linbin))

y2=rbinom(n,1,pro)

y=y1*y2

xn=x1[which(y!=0),]

yn=y[which(y!=0)]

data1=cbind(yn,xn)

data1=data.frame(data1)

gfit1 = glm(yn ~ .,family = gaussian,data1)

cof1=gfit1$coefficients

fa1=1/abs(cof1)

fit1 = glmnet(xn, yn,family='gaussian',penalty.factor=fa1[-1])

#print(fit1)

#plot(fit1)

cv.fit1=cv.glmnet(xn,yn,family='gaussian',type.measure="mse",penalty.factor=fa1[-1])

#plot(cv.fit1)

cv.fit1$lambda.min

SPE1=min(cv.fit1$cvm)

TSPE1=SPE1+TSPE1

coefficients1<-coef(fit1,s=cv.fit1$lambda.min)

coefficients1=coefficients1[-1]

Active.Index1<-which(coefficients1!=0)

nActive1=Active.Index1

Active.coefficients1<-coefficients1[Active.Index1]

SEn1=sum((Beta[nActive1]-Active.coefficients1)^2)

zActive.Index1<-which(coefficients1==0)

zActive1<-zActive.Index1

SEz1=sum((Beta[zActive1])^2)

SE1=(SEn1+SEz1)/p

TSE1=TSE1+SE1

ln1=intersect(nBeta, nActive1)

lz1=intersect(zBeta, zActive1)

Sen1=length(ln1)/length(nBeta)

Spe1=length(lz1)/length(zBeta)

TSen1=Sen1+TSen1

TSpe1=Spe1+TSpe1

Acc1=(length(ln1)+length(lz1))/p

TAcc1=Acc1+TAcc1

data2=cbind(y2,x1)

data2=data.frame(data2)

gfit2 = glm(y2 ~ .,family =binomial(link = "logit") ,data2)

cof2=gfit2$coefficients

fa2=1/abs(cof2)

fit2 = glmnet(x1, y2,family='binomial',penalty.factor=fa2[-1])

#print(fit2)

#plot(fit2)

cv.fit2=cv.glmnet(x1,y2,family='binomial',type.measure="deviance",penalty.factor=fa2[-1])

#plot(cv.fit2)

cv.fit2$lambda.min

SPE2=min(cv.fit2$cvm)

TSPE2=SPE2+TSPE2

coefficients2<-coef(fit2,s=cv.fit2$lambda.min)

coefficients2=coefficients2[-1]

Active.Index2<-which(coefficients2!=0)

nActive2=Active.Index2

Active.coefficients2<-coefficients2[Active.Index2]

SEn2=sum((Gamma[nActive2]-Active.coefficients2)^2)

zActive.Index2<-which(coefficients2==0)

zActive2=zActive.Index2

SEz2=sum((Gamma[zActive2])^2)

SE2=(SEn2+SEz2)/p

TSE2=SE2+TSE2

ln2=intersect(nGamma, nActive2)

lz2=intersect(zGamma, zActive2)

Sen2=length(ln2)/length(nGamma)

Spe2=length(lz2)/length(zGamma)

TSen2=Sen2+TSen2

TSpe2=Spe2+TSpe2

Acc2=(length(ln2)+length(lz2))/p

TAcc2=Acc2+TAcc2

SPE=SPE1+SPE2

TSPE=SPE+TSPE

SE=(SE1+SE2)/2

TSE=SE+TSE

Sen=(length(ln1)+length(ln2))/(length(nBeta)+length(nGamma))

TSen=Sen+TSen

Spe=(length(lz1)+length(lz2))/(length(zBeta)+length(zGamma))

TSpe=Spe+TSpe

Acc=(length(ln1)+length(ln2)+length(lz1)+length(lz2))/(p+p)

TAcc=Acc+TAcc

}

TSPE1/500

TSE1/500

TSen1/500

TSpe1/500

TAcc1/500

TSPE2/500

TSE2/500

TSen2/500

TSpe2/500

TAcc2/500

TSPE/500

TSE/500

TSen/500

TSpe/500

TAcc/500

**3. Empirical part (Lasso variable selection)**

library("glmnet")

X0=read.csv("E:\\WPFHEICHILD-Research-All.csv")

X1=X0[X0$visit_nbr=="V01",]

c=c(5,6,11,16)

X2=X1[,c]

X3=log(X2)

X3[sapply(X3,is.infinite)]=0

X4=X3

X4[X4$F_TOTAL!=0,1]=1

X4[X4$F_WHOLE!=0,2]=1

X4[X4$V_DOL!=0,3]=1

X4[X4$GRAIN_WHOLE!=0,4]=1

FT=X3$F_TOTAL

FT1=FT[which(FT!=0)]

shapiro.test(FT1)

FW=X3$F_WHOLE

FW1=FW[which(FW!=0)]

shapiro.test(FW1)

VD=X3$V_DOL

VD1=VD[which(VD!=0)]

shapiro.test(VD1)

GW=X3$GRAIN_WHOLE

GW1=GW[which(GW!=0)]

shapiro.test(GW1)

V0=read.csv("E:\\V01-surveyeatingandbiomed-short.csv")

V1=V0[,-c(1,2,5,6,7,8,9,10,11,38,39,40,41,42)]

D1=cbind(X4,V1)

D2=cbind(X3,V1)

FTD=D1[,-c(2,3,4)]

fit1 = glmnet(as.matrix(D1[,-c(1,2,3,4)]), D1$F_TOTAL,family='binomial')

#print(fit2)

#plot(fit2)

cv.fit1=cv.glmnet(as.matrix(D1[,-c(1,2,3,4)]),D1$F_TOTAL,family='binomial',type.measure="deviance")

cv.fit1$lambda.min

SPE1=min(cv.fit1$cvm)

coefficients1<-coef(fit1,s=cv.fit1$lambda.min)

Active.Index1<-which(coefficients1!=0)

Active.coefficients1<-coefficients1[Active.Index1]

zActive.Index1<-which(coefficients1==0)

VDD=D1[,-c(2,3,4)]

fit3 = glmnet(as.matrix(D1[,-c(1,2,3,4)]), D1$V_DOL,family='binomial')

#print(fit2)

#plot(fit2)

cv.fit3=cv.glmnet(as.matrix(D1[,-c(1,2,3,4)]),D1$V_DOL,family='binomial',type.measure="deviance")

cv.fit3$lambda.min

SPE3=min(cv.fit3$cvm)

#cv.fit1$lambda.1se

coefficients3<-coef(fit3,s=cv.fit3$lambda.min)

Active.Index3<-which(coefficients3!=0)

Active.coefficients3<-coefficients3[Active.Index3]

zActive.Index3<-which(coefficients3==0)

l=as.matrix(D1[,-c(1,2,3,4)])

l1=l%*%coefficients3[-1]+coefficients3[1]

m1=D1$V_DOL*l1

m2=log(1+exp(l1))

lik1=sum(l1)-sum(m2)

-2*lik1

2*(length(Active.Index3)-1)-2*lik1

(length(Active.Index3)-1)*log(136)-2*lik1

VDCD=D2[which(D2$V_DOL!=0),]

cfit1 = glmnet(as.matrix(VDCD[,-c(1,2,3,4)]), VDCD$V_DOL,family='gaussian')

#print(fit1)

#plot(fit1)

ccv.fit1=cv.glmnet(as.matrix(VDCD[,-c(1,2,3,4)]),VDCD$V_DOL,family='gaussian',type.measure="mse")

#plot(cv.fit1)

ccv.fit1$lambda.min

SPE1=min(ccv.fit1$cvm)

ccoefficients1<-coef(cfit1,s=ccv.fit1$lambda.min)

cActive.Index1<-which(ccoefficients1!=0)

cActive.coefficients1<-ccoefficients1[cActive.Index1]

czActive.Index1<-which(ccoefficients1==0)

p=predict(cfit1, as.matrix(VDCD[,-c(1,2,3,4)]), s = ccv.fit1$lambda.min)

d=as.matrix(VDCD[,-c(1,2,3,4)])

d1=d%*%ccoefficients1[-1]+ccoefficients1[1]

a1=(VDCD$V_DOL-d1)^2/(2*(SPE1))

a2=log(SPE1)/2

a3=log(2*pi)/2

lik2=nrow(d)*(-a2-a3)-sum(a1)

-2*lik2#-2log

2*(length(cActive.Index1)-1)-2*lik2 #AIC

(length(cActive.Index1)-1)*log(90)-2*lik2 #BIC

-2*log(exp(lik1)*exp(lik2))

2*(length(Active.Index3)+length(cActive.Index1)-2)-2*log(exp(lik1)*exp(lik2))#AIC

(length(Active.Index3)+length(cActive.Index1)-2)*log(136)-2*log(exp(lik1)*exp(lik2)) #BIC

**4. Empirical part (ALasso variable selection)**

library("glmnet")

X0=read.csv("E:\\WPFHEICHILD-Research-All.csv")

X1=X0[X0$visit_nbr=="V01",]

c=c(5,6,11,16)

X2=X1[,c]

X3=log(X2)

X3[sapply(X3,is.infinite)]=0

X4=X3

X4[X4$F_TOTAL!=0,1]=1

X4[X4$F_WHOLE!=0,2]=1

X4[X4$V_DOL!=0,3]=1

X4[X4$GRAIN_WHOLE!=0,4]=1

V0=read.csv("E:\\V01-surveyeatingandbiomed-short.csv")

V1=V0[,-c(1,2,5,6,7,8,9,10,11,38,39,40,41,42)]

D1=cbind(X4,V1)

D2=cbind(X3,V1)

vdD1=D1[,-c(1,2,4)]

fit1 = glm(V_DOL ~ .,family =binomial(link = "logit") ,vdD1)

cof1=fit1$coefficients

fa1=1/abs(cof1)

fit2 = glmnet(as.matrix(D1[,-c(1,2,3,4)]), D1$V_DOL,family='binomial',penalty.factor=fa1[-1])

cv.fit1=cv.glmnet(as.matrix(D1[,-c(1,2,3,4)]), D1$V_DOL,family='binomial',type.measure="deviance",penalty.factor=fa1[-1])

#plot(cv.fit2)

cv.fit1$lambda.min

SPE2=min(cv.fit1$cvm)

coefficients1<-coef(fit2,s=cv.fit1$lambda.min )

Active.Index1<-which(coefficients1!=0)

Active.coefficients1<-coefficients1[Active.Index1]

zActive.Index1<-which(coefficients1==0)

l=as.matrix(D1[,-c(1,2,3,4)])

l1=l%*%coefficients1[-1]+coefficients1[1]

m1=D1$V_DOL*l1

m2=log(1+exp(l1))

lik1=sum(l1)-sum(m2)

-2*lik1

2*(length(Active.Index1)-1)-2*lik1

(length(Active.Index1)-1)*log(136)-2*lik1

VDCD=D2[which(D2$V_DOL!=0),]

VDCD1=VDCD[,-c(1,2,4)]

cfit1 = glm(V_DOL ~ .,family = gaussian,VDCD1)

ccof1=cfit1$coefficients

cfa1=1/abs(ccof1)

cfit2 = glmnet(as.matrix(VDCD[,-c(1,2,3,4)]), VDCD$V_DOL,family='gaussian',penalty.factor=cfa1[-1])

#print(fit1)

#plot(fit1)

ccv.fit1=cv.glmnet(as.matrix(VDCD[,-c(1,2,3,4)]), VDCD$V_DOL,family='gaussian',type.measure="mse",penalty.factor=cfa1[-1])

#plot(cv.fit1)

ccv.fit1$lambda.min

SPE1=min(ccv.fit1$cvm)

ccoefficients1<-coef(cfit2,s=ccv.fit1$lambda.min)

cActive.Index1<-which(ccoefficients1!=0)

cActive.coefficients1<-ccoefficients1[cActive.Index1]

czActive.Index1<-which(ccoefficients1==0)

d=as.matrix(VDCD[,-c(1,2,3,4)])

d1=d%*%ccoefficients1[-1]+ccoefficients1[1]

a1=(VDCD$V_DOL-d1)^2/(2*(SPE1))

a2=log(SPE1)/2

a3=log(2*pi)/2

lik2=nrow(d)*(-a2-a3)-sum(a1)

-2*lik2

2*(length(cActive.Index1)-1)-2*lik2

(length(cActive.Index1)-1)*log(90)-2*lik2

-2*log(exp(lik1)*exp(lik2))

2*(length(Active.Index1)+length(cActive.Index1)-2)-2*log(exp(lik1)*exp(lik2))

(length(Active.Index1)+length(cActive.Index1)-2)*log(136)-2*log(exp(lik1)*exp(lik2))
