# Supplementary material for: The variable selection of two-part regression model for semicontinuous data
Source: PLoS One. 2025 Jun 3;20(6):e0322937. doi: 10.1371/journal.pone.0322937 (PMC12132987; doi:10.1371/journal.pone.0322937)
Supplement: Attachment — Submitted filename: comments - PONE-D-24-37521.pdf [file pone.0322937.s002.pdf]

The Variable Selection of Two-part Regression Model for Semicontinuous Data

by Yahui Lu, Aiyi Liu and Tao Jiang

The paper introduces the Bernoulli-Normal two-part (BNT) regression model and proposes a new type of variable selection method based on Lasso penalty function. To solve the problem that Lasso estimator does not have Oracle attribute, the paper also proposes a variable selection method based on adaptive Lasso penalty function.

The study presents the results of original research and have not been published elsewhere. The article is well-written, and the addressed problem is highly relevant. The title effectively conveys the content of the article. The abstract appropriately synthesizes the text, and the keywords used are precise. Furthermore, conclusions are presented in an appropriate fashion and are supported by the data.

I recommend the paper for publication. I suggest that the authors specify which software was used to conduct the analyses. Additionally, if feasible, making the code for the simulations and applications available to readers would enhance the paper's transparency and reproducibility.
