# Supplementary material for: The variable selection of two-part regression model for semicontinuous data
Source: PLoS One. 2025 Jun 3;20(6):e0322937. doi: 10.1371/journal.pone.0322937 (PMC12132987; doi:10.1371/journal.pone.0322937)
Supplement: Attachment — Submitted filename: Response to Reviewers.docx [file pone.0322937.s003.docx]

**Response to Comments from Reviewers**

Dear Editors and Reviewers,

We sincerely thank you for your careful reading of our manuscript and for providing constructive comments. We have carefully taken into consideration the comments from you. Accordingly, we have made substantial changes based on these comments and provided detailed point-by-point responses.

Below are our point-by-point responses to your specific concerns. For your convenience, changes made accordingly are highlighted in the revised manuscript.

*Editors’ Comments*

1. *Please ensure that your manuscript meets PLOS ONE's style requirements, including those for file naming.*

**Response:** Many thanks for your kind help in dealing with our paper. In this revision, we have made changes to the title font size, author information and unit information, and corresponding authors involved in this paper according to the requirements of the journal.

2. *Please update your submission to use the PLOS LaTeX template.*

**Response:** Many thanks for your kind help in dealing with our paper. In this revision, we have updated according to the PLOS LaTeX template.

3. *Please note that PLOS ONE has specific guidelines on code sharing for submissions in which author-generated code underpins the findings in the manuscript. In these cases, we expect all author-generated code to be made available without restrictions upon publication of the work.*

**Response:** Many thanks for your kind help in dealing with our paper. In this revision, we have organized the main code of the paper and made it available upon request, ensuring that the code is shared in a way that follows best practices and promotes reproducibility and reuse.

4. *Thank you for stating the following financial disclosure: [Research of A. Liu is supported by the Intramural Research Program of the Eunice Kennedy Shriver National Institute of Child Health and Human Development. Lu’s work is supported by the research foundation of Zhejiang University of Science and Technology (2023QN094) and Hangzhou philosophy and social science planning project (Z23JC042).]. Please state what role the funders took in the study. If the funders had no role, please state: "The funders had no role in study design, data collection and analysis, decision to publish, or preparation of the manuscript." If this statement is not correct you must amend it as needed. Please include this amended Role of Funder statement in your cover letter; we will change the online submission form on your behalf.*

**Response:** Many thanks for your kind help in dealing with our paper. In this revision, we have re-added funders and stated what role the funders took in the study within the Acknowledgments Section. See below and highlights.

In the cover letter, we also added this amended Role of Funder statement.

**“This work was supported by the Research Project of Zhejiang Federation of Humanities and Social Sciences [No.2025N072], and in part by the Hangzhou Philosophy and Social Science Planning Project [No.Z23JC042]. The funders all played an important role in the study research design, data collection and analysis. The authors were grateful to the referees, associate editor, and the editor for their valuable comments and suggestions. The authors thanked Dr. Tonja Nansel for helpful discussions on the CHEF study.”**

*Response to Reviewer:*

*Reviewer #1:* *The study presents the results of original research and have not been published else-where. The article is well-written, and the addressed problem is highly relevant. The title effectively conveys the content of the article. The abstract appropriately synthesizes the text, and the keywords used are precise. Furthermore, conclusions are presented in an appropriate fashion and are supported by the data. I recommend the paper for publication.*

**Response:** Thank you for your positive assessment.

*1. The data was not made available. Also, I suggest that the authors specify which software was used to* *conduct the analyses. Additionally, if feasible, making the code for the simulations and applications available to readers would enhance the paper's transparency and reproducibity.*

**Response:** Point well taken. All the code in this paper was carried out in R software, and we have provided all the code in the Appendix. We have added some more descriptions in Section 1 of our manuscript. See below and highlights.

**“All simulations and real data analysis results are conducted by R software, and all codes have been provided in the Appendix (See S1 Appendix).”**

The application CHEF data was mainly provided by Professor Aiyi Liu of the Eunice Kennedy Shriver National Institute of Child Health and Human Development, and we would like to sincerely thank Dr.Nansel for the detailed introduction of the data. As the data is not yet available to the public, please forgive us that we cannot provide the detailed URL of the data source. However, Nansel et al. (2015) introduced the specific design, randomization procedure and treatment conditions of this study in detail (**see reference [23]**). In the Appendix, we have provided the detailed code for the reviewer or other scholars, and the parameters can be appropriately adjusted according to their own research content.

*Reviewer #2: In this paper, authors proposed methods for variable selection for the two-part regression model specifically, the Bernoulli-Normal two-part (BNT) regression model. The variable selection method is based on adaptive Lasso penalty function. The following are detailed comments:*

**Response:** We are very much grateful to you for your careful reading of our manuscript and for your constructive comments. The manuscript has been revised based on your valuable comments and suggestions. In response to your thoughtful and insightful comments and suggestions, we conducted additional simulation studies to prove the performance of the proposed method. Details on the set-up of the simulations are given in the Appendix.

*1. The usage of normal model for the non-zero part is limited. In reality, often the continuous measure does not follow normal distribution.*

**Response:** Thanks for the thoughtful comment. This is a good question and could be an issue with any two-part models of semicontinuous data. For any two-part models, its performance usually depends on the assumptions made on the model. If these assumptions are violated, the model may well be under performance. In our manuscript, we focus on the variable selection problem of the Bernoulli-Normal model for semicontinuous data. The presence/absence of zeros is assumed to follow a Bernoulli distribution. The nonzero values are assumed to follow a normal distribution. When the continuous part of the distribution happens to be non-normal, the variable selection performance of the Lasso and the adaptive Lasso may be affected to some extent. At this time, the proposed methods can be extended or improved by making reasonable assumptions about the distribution of continuous data. This is also one of our future research directions. We have added some more descriptions in Section 6 of our manuscript. See below and highlights.

**“Considering the distribution characteristics of the continuous part of the semicontinuous data, different two-part models can be constructed, such as the Bernoulli-Gamma model and Bernoulli-Skewed Normal model. The variable selection problem of these regression models is one of the subsequent research problems.”**

*2. It would be interesting to see the model performance for the following scenarios*

*a)* *Varying Zero Proportions including low, moderate, and high zero proportions*

**Response:** Point well taken. We conduct more simulations following your comments, by generating covariables from the multivariate normal distribution$N_{q}(0,\Sigma)$, where covariance matrix is Σ whose elements are $\rho^{|i-j|}(i,j=1,\ldots,q)$. In the section, we set $\rho=0.6$, $q=15$, and the sample size is $n=300$. According to the Bernoulli-Normal regression model, the real model is assumed as follows, where $\text{logit}(\pi_{i})=\mathbf{z}_{1}^{T}\mathbf{\beta}_{1}$, $\mu_{i}=\mathbf{z}_{2}^{T}\mathbf{\beta}_{2}$. We set $\mathbf{z}_{1}=\mathbf{z}_{2}$ in the following simulation scenarios, and each scenario is repeated 500 times. In this section, the model coefficient settings include different zero proportions, including low, moderate, and high zero proportions.

(1) When $\Pr(\beta=0)=0.3$, the regression coefficients in model are set as

(2) When $\Pr(\beta=0)=0.6$, the regression coefficients in model are set as

(3) When $\Pr(\beta=0)=0.8$, the regression coefficients in model are set as

$\mathbf{\beta}_{1}=(0.2,0.30,1,0,0.4,0,0,0,0,0,0,0,0,0,0,0)^{T}$,

$$\mathbf{\beta}_{2}=(0.1,1.10,1,-0.36,0,0,0,0,0,0,0,0,0,0,0,0)^{T}.$$

**Table 1.** Simulation results of the setting $\Pr(\beta=0)=0.3$

| **Method** | **MPSE** | **MSE** | **Sensitivity** | **Specificity** | **Accuracy** |
| --- | --- | --- | --- | --- | --- |
| Binomial Part |  |  |  |  |  |
| LASSO | 0.7982 | 0.0552 | 0.9332 | 0.4636 | 0.7767 |
| ALASSO | 0.7799 | 0.0605 | 0.8450 | 0.7448 | 0.8116 |
| Normal Part |  |  |  |  |  |
| LASSO | 1.1203 | 0.0149 | 0.9970 | 0.2960 | 0.7633 |
| ALSSO | 1.0888 | 0.0134 | 0.9902 | 0.7244 | 0.9016 |
| Two Parts |  |  |  |  |  |
| LASSO | 1.9184 | 0.0350 | 0.9651 | 0.3798 | 0.7700 |
| ALSSO | 1.8686 | 0.0370 | 0.9176 | 0.7346 | 0.8566 |

**Table 2.** Simulation results of the setting $\Pr(\beta=0)=0.6$

| **Method** | **MPSE** | **MSE** | **Sensitivity** | **Specificity** | **Accuracy** |
| --- | --- | --- | --- | --- | --- |
| Binomial Part |  |  |  |  |  |
| LASSO | 1.0906 | 0.0123 | 0.9644 | 0.5122 | 0.6629 |
| ALASSO | 1.0382 | 0.0088 | 0.9784 | 0.7754 | 0.8431 |
| Normal Part |  |  |  |  |  |
| LASSO | 0.9359 | 0.0265 | 0.8433 | 0.6469 | 0.7255 |
| ALSSO | 0.9235 | 0.0337 | 0.7517 | 0.7842 | 0.7712 |
| Two Parts |  |  |  |  |  |
| LASSO | 2.0265 | 0.0194 | 0.8984 | 0.5760 | 0.6942 |
| ALSSO | 1.9617 | 0.0213 | 0.8547 | 0.7796 | 0.8071 |

**Table 3.** Simulation results of the setting $\Pr(\beta=0)=0.8$

| **Method** | **MPSE** | **MSE** | **Sensitivity** | **Specificity** | **Accuracy** |
| --- | --- | --- | --- | --- | --- |
| Binomial Part |  |  |  |  |  |
| LASSO | 1.1083 | 0.0129 | 0.9393 | 0.7367 | 0.7772 |
| ALASSO | 1.0895 | 0.0183 | 0.8160 | 0.8135 | 0.8140 |
| Normal Part |  |  |  |  |  |
| LASSO | 1.0628 | 0.0080 | 0.9707 | 0.6285 | 0.6969 |
| ALSSO | 1.0240 | 0.0064 | 0.9740 | 0.8098 | 0.8427 |
| Two Parts |  |  |  |  |  |
| LASSO | 2.1711 | 0.0104 | 0.9550 | 0.6826 | 0.7371 |
| ALSSO | 2.1135 | 0.0124 | 0.8950 | 0.8117 | 0.8283 |

Table 1-3 above present the simulation results. From the tables, the two proposed methods have good variable selection effect for different zero proportions, and the adaptive Lasso method is superior to Lasso method.

We present the results in the Appendix.

*b)* *Different Variable-Effect Strengths including strong and weak signal between predictors and outcome in one or both parts of the model.*

**Response:** Point well taken. In linear regression, it is necessary to check the effect strength of different variables (independent variables) on dependent variables, and the regression coefficient can directly reflect the influence degree of independent variables on dependent variables. If the regression coefficient is positive, it means that the dependent variable increases with the increase of the independent variable. If the regression coefficient is negative, it means that the dependent variable decreases as the independent variable increases. The greater the absolute value of the regression coefficient, the more significant the influence of the independent variable on the dependent variable‌.

To observe different Variable-Effect strengths including strong and weak signal between predictors and outcome, we conduct simulation studies by setting different regression coefficient values, so as to further verify the variable selection effect of our proposed methods in the Bernoulli-Normal model. In the study, covariables are generated from the multivariate normal distribution $N_{q}(0,\Sigma)$, where covariance matrix is Σ whose elements are $\rho^{|i-j|}(i,j=1,\ldots,q)$. In the settings above, the sample size is $n=300$, $\rho=0,0.6$, $q=15$. We set $\mathbf{z}_{1}=\mathbf{z}_{2}$ in the following simulation scenarios.

(1) When there is a weak Variable-Effect strength between the predictor and the outcome, the regression coefficients are set to

(2) When there is a strong Variable-Effect strength between the predictor and the outcome, the regression coefficients are set to

$$\mathbf{\beta}_{1}=(1.2,1.2,1.2,1.2,1.2,-1.2,0,0,1.2,0,0,0,0,0,0,0)^{T}\text{,}$$

$$\mathbf{\beta}_{2}=(1,1,1,-1,1,0,0,0,1,0,0,0,0,0,0,0)^{T}.$$

Table S4 - S7 below present the simulation results. From the Table 4 and Table 5, the proposed methods have high Specificity value and low Sensitivity value. This is because the strength of the weak variable effect between the predictor and the outcome results in more variable coefficients being estimated at zero. On the contrary, from the Table 6 and Table 7, the proposed methods have high Sensitivity value and low Specificity value, with the Sensitivity value mostly approaching 1. This is because the strength of the strong variable effect between the predictor and the outcome results in more variable coefficients being estimated at nonzero, thus classifying more variables as important. However, all the results show that the proposed adaptive Lasso method has higher Sensitivity value, Specificity value and Accuracy value than the Lasso method. Therefore, the adaptive lasso method has a better variable selection effect for the Bernoulli-Normal model.

We present the results in the Appendix.

**Table 4.** Simulation results of the weak Variable-Effect ($\rho=0$)

| **Method** | **MPSE** | **MSE** | **Sensitivity** | **Specificity** | **Accuracy** |
| --- | --- | --- | --- | --- | --- |
| Binomial Part |  |  |  |  |  |
| LASSO | 1.3638 | 0.0105 | 0.6113 | 0.7227 | 0.6781 |
| ALASSO | 1.3501 | 0.0111 | 0.6537 | 0.7622 | 0.7188 |
| Normal Part |  |  |  |  |  |
| LASSO | 1.0295 | 0.0034 | 0.3512 | 0.8128 | 0.6589 |
| ALSSO | 1.0201 | 0.0043 | 0.4256 | 0.7902 | 0.6687 |
| Two Parts |  |  |  |  |  |
| LASSO | 2.3923 | 0.0070 | 0.4931 | 0.7701 | 0.6685 |
| ALSSO | 2.3703 | 0.0077 | 0.5500 | 0.7769 | 0.6937 |

**Table 5.** Simulation results of the weak Variable-Effect ($\rho=0.6$)

| **Method** | **MPSE** | **MSE** | **Sensitivity** | **Specificity** | **Accuracy** |
| --- | --- | --- | --- | --- | --- |
| Binomial Part |  |  |  |  |  |
| LASSO | 1.3281 | 0.0125 | 0.5080 | 0.7808 | 0.6717 |
| ALASSO | 1.3186 | 0.0178 | 0.6130 | 0.7604 | 0.7015 |
| Normal Part |  |  |  |  |  |
| LASSO | 1.0353 | 0.0037 | 0.3528 | 0.8064 | 0.6552 |
| ALSSO | 1.0138 | 0.0052 | 0.3072 | 0.8374 | 0.6607 |
| Two Parts |  |  |  |  |  |
| LASSO | 2.3634 | 0.0081 | 0.4375 | 0.7943 | 0.6635 |
| ALSSO | 2.3325 | 0.0115 | 0.4740 | 0.8009 | 0.6811 |

**Table 6.** Simulation results of the strong Variable-Effect ($\rho=0$)

| **Method** | **MPSE** | **MSE** | **Sensitivity** | **Specificity** | **Accuracy** |
| --- | --- | --- | --- | --- | --- |
| Binomial Part |  |  |  |  |  |
| LASSO | 1.7752 | 0.0386 | 1.0000 | 0.3867 | 0.6321 |
| ALASSO | 1.7580 | 0.0257 | 1.0000 | 0.8264 | 0.8959 |
| Normal Part |  |  |  |  |  |
| LASSO | 1.0935 | 0.0064 | 1.0000 | 0.4880 | 0.6587 |
| ALSSO | 1.0454 | 0.0043 | 1.0000 | 0.8392 | 0.8928 |
| Two Parts |  |  |  |  |  |
| LASSO | 1.8686 | 0.0225 | 1.0000 | 0.4401 | 0.6454 |
| ALSSO | 1.8034 | 0.0150 | 1.0000 | 0.8332 | 0.8943 |

**Table 7.** Simulation results of the strong Variable-Effect ($\rho=0.6$)

| **Method** | **MPSE** | **MSE** | **Sensitivity** | **Specificity** | **Accuracy** |
| --- | --- | --- | --- | --- | --- |
| Binomial Part |  |  |  |  |  |
| LASSO | 0.6729 | 0.0621 | 1.0000 | 0.4816 | 0.6889 |
| ALASSO | 0.6632 | 0.0527 | 0.9993 | 0.7796 | 0.8675 |
| Normal Part |  |  |  |  |  |
| LASSO | 1.0809 | 0.0113 | 1.0000 | 0.4836 | 0.6557 |
| ALSSO | 1.0404 | 0.0077 | 1.0000 | 0.8240 | 0.8827 |
| Two Parts |  |  |  |  |  |
| LASSO | 1.7538 | 0.0367 | 1.0000 | 0.4826 | 0.6723 |
| ALSSO | 1.7037 | 0.0302 | 0.9996 | 0.8029 | 0.8751 |

*c)* *Different Correlations Between Parts. The current simulation assumes independent parts. However, it will be useful to test the robustness of the model under the violation of the independence assumption.*

**Response:** Point well taken. In this paper, we seek a feasible way of conducting variable selection for the Bernoulli-Normal model. Based on the basic idea of constructing a two-part model, we divide the model into two parts. For the first part, whether *X* is zero can be treated as being from a Bernoulli distribution, that is, it is assumed that *Y* follows a Bernoulli distribution. For the second part, the nonzero part is assumed to follow a normal distribution. The model assumes that the two parts are independent. But when the two parts do not satisfy the independence, then the model becomes a random effects Bernoulli-Normal model, that is

$f(x_{i})=(1-\pi_{i})^{1-y_{i}}[\pi_{i}N(x_{i};\mu_{i},\sigma^{2})]^{y_{i}}$,

$\text{logit}(\pi_{i})=\text{logit}[\text{Pr}(X_{i}>0)]=\mathbf{z}_{1i}^{T}\mathbf{\beta}_{1}+u_{1i}$,

$\mu_{i}=\text{E}[X_{i}|X_{i}>0]=\mathbf{z}_{2i}^{T}\mathbf{\beta}_{2}+u_{2i}$.

The random effects $u_{1i}$ and $u_{2i}$ from the two parts are assumed to be jointly normally distributed and possibly correlated

$\left(\begin{matrix} u_{1i} \\ u_{2i} \end{matrix}\right)\sim N\left(\left[\begin{matrix} 0 \\ 0 \end{matrix}\right],\left[\begin{matrix} \sigma_{1} & \sigma_{12} \\ \sigma_{12} & \sigma_{2} \end{matrix}\right]\right)$.

In this section, in order to verify the variable selection effect of our proposed methods in the random effects Bernoulli-Normal model, we conduct simulation studies to examine the finite sample performance of the model. In the study, covariables are generated from the multivariate normal distribution $N_{q}(0,\Sigma)$, where covariance matrix is Σ whose elements are $\rho^{|i-j|}(i,j=1,\ldots,q)$. In the settings above, the sample size is $n=300$, $\rho=0,0.6$, $q=15$, $\left(\sigma_{1},\sigma_{2},\sigma_{12}\right)=\left(1,1.44,0.6\right)$. In the following simulation scenarios, we set $\mathbf{z}_{1}=\mathbf{z}_{2}$ and the regression coefficients in model are set as

$$\mathbf{\beta}_{1}=(0.2,0.30,1,0.8,0.4,-0.2,0,0,0.6,0,0,0,0,0,0,0)^{T}\text{,}$$

$$\mathbf{\beta}_{2}=(0.1,1.10,1,-0.36,0.6,0,0,0,0.8,0,0,0,0,0,0,0)^{T}.$$

Table 8 and Table 9 below present the simulation results. From the tables, under the violation of the independence assumption between parts, the two proposed methods also have good effect for the variable selection of the random effects Bernoulli-Normal model, and the adaptive Lasso method is superior to Lasso method. However, compared with the Bernoulli-Normal model, the proposed methods have higher prediction error in the random effects Bernoulli-Normal model. Because fitting a random effects two-part model itself faces substantial computational challenges, which mostly arise from numerical integration with respect to correlated random effects.

We present the results in the Appendix.

**Table 8.** Simulation results of the random effects Bernoulli-Normal model ($\rho=0$)

| **Method** | **MPSE** | **MSE** | **Sensitivity** | **Specificity** | **Accuracy** |
| --- | --- | --- | --- | --- | --- |
| Binomial Part |  |  |  |  |  |
| LASSO | 1.1802 | 0.0313 | 0.9067 | 0.5736 | 0.7068 |
| ALASSO | 1.1649 | 0.0253 | 0.8390 | 0.7902 | 0.8097 |
| Normal Part |  |  |  |  |  |
| LASSO | 1.1395 | 0.0136 | 0.9992 | 0.4868 | 0.6576 |
| ALSSO | 2.0469 | 0.0106 | 0.9944 | 0.7840 | 0.8541 |
| Two Parts |  |  |  |  |  |
| LASSO | 3.3197 | 0.0225 | 0.9487 | 0.5279 | 0.6822 |
| ALSSO | 3.2118 | 0.0180 | 0.9096 | 0.7869 | 0.8319 |

**Table 9.** Simulation results of the random effects Bernoulli-Normal model ($\rho=0.6$)

| **Method** | **MPSE** | **MSE** | **Sensitivity** | **Specificity** | **Accuracy** |
| --- | --- | --- | --- | --- | --- |
| Binomial Part |  |  |  |  |  |
| LASSO | 1.0367 | 0.0367 | 0.8140 | 0.6573 | 0.7200 |
| ALASSO | 1.0200 | 0.0371 | 0.7120 | 0.7960 | 0.7624 |
| Normal Part |  |  |  |  |  |
| LASSO | 2.1315 | 0.0239 | 0.9364 | 0.5440 | 0.6748 |
| ALSSO | 2.0363 | 0.0201 | 0.9456 | 0.7590 | 0.8212 |
| Two Parts |  |  |  |  |  |
| LASSO | 3.1682 | 0.0303 | 0.8696 | 0.5977 | 0.6974 |
| ALSSO | 3.0563 | 0.0286 | 0.8182 | 0.7765 | 0.7918 |

*d) It is not clear* *the model performance with high-dimensional data. The current simulation assumes the largest number of covariates in the model is 25.*

**Response:** Thanks for the very thoughtful comment! We agree that the number of covariates in the current simulation are relatively small, mainly to be closer to the situation of the example data in this paper. Following your comments, we conduct more simulations by generated covariables from the multivariate normal distribution $N_{q}(0,\Sigma)$, where covariance matrix is $\Sigma$ whose elements are$\rho^{|i-j|}(i,j=1,\ldots,q)$ with $q=100, \rho=0.6$, and total sample sizes is $n=300$. In the following simulation scenario, we set $\mathbf{z}_{1}=\mathbf{z}_{2}$ and the regression coefficients are set as

$$\begin{matrix} \mathbf{\beta}_{1}=(0.2,0.30,1,0.8,0.4,-0.2,0,0,0.6,0,0,0,0,0,0,0,1.1,-0.2,0,0,0,0,0,0.6,0,0\text{，} \\ \text{ }0.30,1,0.8,0.4,-0.2,0,0,0.6,0,0,0,0,0,0,0,1.1,-0.2,0,0,0,0,0,0.6,0,0\text{，} \\ \text{ }0.30,1,0.8,0.4,-0.2,0,0,0.6,0,0,0,0,0,0,0,1.1,-0.2,0,0,0,0,0,0.6,0,0\text{，} \\ \text{ }0.30,1,0.8,0.4,-0.2,0,0,0.6,0,0,0,0,0,0,0,1.1,-0.2,0,0,0,0,0,0.6,0,0)^{T}\text{,} \end{matrix}$$

$$\begin{matrix} \mathbf{\beta}_{2}=(0.1,1.10,1,-0.36,0.6,0,0,0,0.8,0,0,0,0,0,0,0,0.9,0.7,0,0,0,0,0,1.2,0,0\text{，} \\ \text{ }1.10,1,-0.36,0.6,0,0,0,0.8,0,0,0,0,0,0,0,0.9,0.7,0,0,0,0,0,1.2,0,0\text{，} \\ \text{ }1.10,1,-0.36,0.6,0,0,0,0.8,0,0,0,0,0,0,0,0.9,0.7,0,0,0,0,0,1.2,0,0\text{，} \\ \text{ }1.10,1,-0.36,0.6,0,0,0,0.8,0,0,0,0,0,0,0,0.9,0.7,0,0,0,0,0,1.2,0,0)^{T} \end{matrix}$$

**Table 10.** Simulation results of the high-dimensional setting

| **Method** | **MPSE** | **MSE** | **Sensitivity** | **Specificity** | **Accuracy** |
| --- | --- | --- | --- | --- | --- |
| Binomial Part |  |  |  |  |  |
| LASSO | 0.6987 | 0.0583 | 0.7361 | 0.6773 | 0.6985 |
| ALASSO | 0.6187 | 0.0833 | 0.7354 | 0.7026 | 0.7174 |
| Normal Part |  |  |  |  |  |
| LASSO | 2.0063 | 0.0165 | 0.9800 | 0.4847 | 0.6432 |
| ALSSO | 1.3562 | 0.0151 | 0.9783 | 0.6842 | 0.7769 |
| Two Parts |  |  |  |  |  |
| LASSO | 2.7050 | 0.0374 | 0.8509 | 0.5781 | 0.6708 |
| ALSSO | 1.9750 | 0.0492 | 0.8346 | 0.6931 | 0.7276 |

The results are repeated 500 times and presented in the Table 10 above. Results from table demonstrate that the adaptive Lasso method performs better than Lasso method for the Bernoulli-Normal model. In addition, our research is mainly for the case of $p\ll n$, when $p\gg n$, the coordinate descent method may take more time to estimate parameters, which is also one of the contents we are currently studying.

We present the results in the Appendix.

*e)* *Simulate scenarios where some predictors only influence the zero part, while others only influence the non-zero part. This will test the method's capacity to select variables that are important for only one part of the model. The current simulation assumes the same covariates for both parts of the model.*

**Response:** Thanks for the thoughtful comment. The current simulation assumes the same covariates for both parts of the model. But some predictors only influence the zero part, and others only influence the non-zero part. In order to simulate the scenarios, we generate data with different covariables of two parts. The covariables of the zero part ($\mathbf{z}_{1}$) are generated from the multivariate normal distribution $N_{15}(0,\Sigma)$, and the covariables of the non-zero part ($\mathbf{z}_{2}$) are generated from the multivariate normal distribution $N_{10}(0,\Sigma)$, where covariance matrix is $\Sigma$ whose elements are$\rho^{|i-j|}(i,j=1,\ldots,q)$. In the settings above, the sample size is $n=300$, $\rho=0,0.6$, and the regression coefficients in model are set as

$\mathbf{\beta}_{1}=(0.2,0.30,1,0.8,0.4,-0.2,0,0,0.6,0,0,0,0,0,0,0)^{T}$;

$\mathbf{\beta}_{2}=(0.1,1.10,1,-0.36,0.6,0,0,0,0,0,0)^{T}$.

The results are repeated 500 times and presented in the Table 11-12 below. The results show that when the covariables of the two parts are different, the proposed methods still have good variable selection effect in the Bernoulli-Normal model. And the adaptive Lasso method performs better than the Lasso method.

We present the results in the Appendix.

**Table 11.** Simulation results of the different covariables setting ($\rho=0$)

| **Method** | **MPSE** | **MSE** | **Sensitivity** | **Specificity** | **Accuracy** |
| --- | --- | --- | --- | --- | --- |
| Binomial Part |  |  |  |  |  |
| LASSO | 1.0904 | 0.0174 | 0.9400 | 0.5227 | 0.6896 |
| ALASSO | 1.0724 | 0.0160 | 0.8760 | 0.7687 | 0.8116 |
| Normal Part |  |  |  |  |  |
| LASSO | 1.0715 | 0.0058 | 0.1000 | 0.4990 | 0.6994 |
| ALSSO | 1.0353 | 0.0047 | 0.9990 | 0.8143 | 0.8882 |
| Two Parts |  |  |  |  |  |
| LASSO | 2.1619 | 0.0127 | 0.9640 | 0.5132 | 0.6935 |
| ALSSO | 2.1076 | 0.0115 | 0.9252 | 0.7869 | 0.8422 |

**Table 12.** Simulation results of the different covariables setting ($\rho=0.6$)

| **Method** | **MPSE** | **MSE** | **Sensitivity** | **Specificity** | **Accuracy** |
| --- | --- | --- | --- | --- | --- |
| Binomial Part |  |  |  |  |  |
| LASSO | 0.9378 | 0.0255 | 0.8430 | 0.6698 | 0.7391 |
| ALASSO | 0.9238 | 0.0345 | 0.7510 | 0.7851 | 0.7715 |
| Normal Part |  |  |  |  |  |
| LASSO | 1.0667 | 0.0135 | 0.9555 | 0.4727 | 0.6658 |
| ALSSO | 1.0315 | 0.0109 | 0.9680 | 0.7540 | 0.8396 |
| Two Parts |  |  |  |  |  |
| LASSO | 2.0045 | 0.0207 | 0.8880 | 0.5909 | 0.7098 |
| ALSSO | 1.9552 | 0.0250 | 0.8378 | 0.7727 | 0.7987 |

*f)* *Simulate cases where the model is misspecified for one or both parts, to test how robust the variable selection method is to model misspecification.*

**Response:** Thanks for the thoughtful comment. To evaluate the relative performance of the Lasso and the adaptive Lasso method when the continuous parts of the distributions are non-normal, we consider the continuous part to follow gamma distributions. At this point, we still use the Bernoulli-Normal model to fit data. To evaluate the performance of the relative performance of the Lasso and the adaptive Lasso methods, the gamma distribution we used is induced by G$(\alpha, \beta)$. In the simulation, we only generate the gamma distributions with $\beta=1$, and total sample sizes is $n=300$. Covariables are generated from the multivariate normal distribution$N_{q}(0,\Sigma)$, where covariance matrix is Σ whose elements are $\rho^{|i-j|}(i,j=1,\ldots,q)$ with $\rho=0,0.6$, $q=15$. Then the real data is generated by, where $\text{logit}(\pi_{i})=\mathbf{z}_{1}^{T}\mathbf{\beta}_{1}$, $\log\alpha_{i}=\mathbf{z}_{2}^{T}\mathbf{\beta}_{2}$. We set covariables $\mathbf{z}_{1}=\mathbf{z}_{2}$, and the regression coefficients in model are set as

$\mathbf{\beta}_{1}=(0.2,0.30,1,0.8,0.4,-0.2,0,0,0.6,0,0,0,0,0,0,0)^{T}$;

$\mathbf{\beta}_{2}=(0.1,1.10,1,-0.36,0.6,0,0,0,0.8,0,0,0,0,0,0,0)^{T}$.

When the continuous part of the actual data follows gamma distributions, but we still use the mis-specified Bernoulli-Normal model to fit data. Table 13-14 present the results of 500 repetitions. The results show that the proposed methods still have good variable selection effect, and the adaptive Lasso method is superior to the Lasso method. But all the proposed methods have high prediction error. This is not surprising since the non-zero part is not given a suitable model to fit.

We present the results in the Appendix.

**Table 13.** Simulation results of the continuous part follow gamma distributions ($\rho=0$)

| **Method** | **MPSE** | **MSE** | **Sensitivity** | **Specificity** | **Accuracy** |
| --- | --- | --- | --- | --- | --- |
| Binomial Part |  |  |  |  |  |
| LASSO | 1.0910 | 0.0168 | 0.9437 | 0.5224 | 0.6909 |
| ALASSO | 1.0767 | 0.0163 | 0.8790 | 0.7791 | 0.8191 |
| Normal Part |  |  |  |  |  |
| LASSO | 106.1605 | 0.6396 | 0.9500 | 0.5224 | 0.6649 |
| ALSSO | 107.1314 | 0.9241 | 0.9200 | 0.7730 | 0.8220 |
| Two Parts |  |  |  |  |  |
| LASSO | 107.2515 | 0.3282 | 0.9465 | 0.5224 | 0.6779 |
| ALSSO | 108.2081 | 0.4702 | 0.8976 | 0.7759 | 0.8205 |

**Table 14.** Simulation results of the continuous part follow gamma distributions ($\rho=0.6$)

| **Method** | **MPSE** | **MSE** | **Sensitivity** | **Specificity** | **Accuracy** |
| --- | --- | --- | --- | --- | --- |
| Binomial Part |  |  |  |  |  |
| LASSO | 0.9383 | 0.0266 | 0.8510 | 0.6536 | 0.7325 |
| ALASSO | 0.9190 | 0.0334 | 0.7547 | 0.7816 | 0.7708 |
| Normal Part |  |  |  |  |  |
| LASSO | 64.0235 | 0.2807 | 0.8772 | 0.5738 | 0.6749 |
| ALSSO | 58.4421 | 0.4029 | 0.8652 | 0.7702 | 0.8019 |
| Two Parts |  |  |  |  |  |
| LASSO | 64.9618 | 0.1536 | 0.8629 | 0.6116 | 0.7037 |
| ALSSO | 59.3611 | 0.2182 | 0.8049 | 0.7756 | 0.7863 |

*3. There is no software available publicly. It is hard for other researchers to implement the proposed approach.*

**Response:** Point well taken. Your comment is a good one, and closely related to a comment from another reviewer, so our responses are the same. The details are as follows. All the code in this paper was carried out in R software, and we have provided all the code in the Appendix. We have added some more descriptions in Section 1 of our manuscript. See below and highlights.

**“All simulations and real data analysis results are conducted by R software, and all codes have been provided in the Appendix (See S1 Appendix).”**

The application CHEF data was mainly provided by Professor Aiyi Liu of the Eunice Kennedy Shriver National Institute of Child Health and Human Development, and we would like to sincerely thank Dr. Nansel for the detailed introduction of the data. As the data is not yet available to the public, please forgive us that we cannot provide the detailed URL of the data source. However, Nansel et al. (2015) introduced the specific design, randomization procedure and treatment conditions of this study in detail (**see reference [23]**). In the Appendix, we have provided the detailed code for the reviewer or other scholars, and the parameters can be appropriately adjusted according to their own research content.
